# Effect of police action on low-barrier substance use disorder service utilization

**DOI:** 10.1186/s12954-022-00668-8

**Published:** 2022-07-29

**Authors:** Karrin Weisenthal, Simeon D. Kimmel, Jessica Kehoe, Marc R. Larochelle, Alexander Y. Walley, Jessica L. Taylor

**Affiliations:** 1 Department of Emergency Medicine, Boston Medical Center, Boston, MA USA; 2 Division of Infectious Disease, School of Medicine and Boston Medical Center, Boston University, Boston, MA USA; 3 Grayken Center for Addiction, Boston Medical Center, Boston, MA USA; 4 Section of General Internal Medicine, School of Medicine and Boston Medical Center, Boston University, 801 Massachusetts Avenue, 2nd Floor, #2109, Boston, MA 02118 USA

**Keywords:** Opioid use disorder, Substance use disorder, Medications for opioid use disorder, Police action, Bridge clinic

## Abstract

**Background:**

Police action can increase risky substance use patterns by people who use drugs (PWUD), but it is not known how increased police presence affects utilization of low-barrier substance use disorder bridge clinics. Increased police presence may increase or decrease treatment-seeking behavior. We examined the association between Operation Clean Sweep (OCS), a 2-week police action in Boston, MA, and visit volume in BMC’s low-barrier buprenorphine bridge clinic.

**Methods:**

In this retrospective cohort, we used segmented regression to investigate whether the increased police presence during OCS was associated with changes in bridge clinic visits. We used General Internal Medicine (GIM) clinic visit volume as a negative control. We examined visits during the 6 weeks prior, 2 weeks during, and 4 weeks after OCS (June 18–September 11, 2019).

**Results:**

Bridge clinic visits were 2.8 per provider session before, 2.0 during, and 3.0 after OCS. The mean number of GIM clinic visits per provider session before OCS was 7.0, 6.8 during, and 7.0 after OCS. In adjusted segmented regression models for bridge clinic visits per provider session, there was a nonsignificant level increase (0.643 *P* = 0.171) and significant decrease in slope (0.100, *P* = 0.045) during OCS. After OCS completed, there was a significant level increase (1.442, *P* = 0.003) and slope increase in visits per provider session (0.141, *P* = 0.007). There was no significant change in GIM clinic volume during the study period.

**Conclusions:**

The increased policing during OCS was associated with a significant decrease in bridge clinic visits. Following the completion of OCS, there was a significant increase in clinic visits, suggesting pent-up demand for medications for opioid use disorder, a life-saving treatment.

## Introduction

Surges in law enforcement activity in areas of concentrated homelessness, drug use, and street-apparent poverty that involve systematic searches, threats, and arrests are known as “crackdowns” or “sweeps” [1–6]. These policing strategies seek to disrupt the drug supply, compel people into addiction treatment, and move people out of specific areas. They also displace PWUD from their usual communities, drug supply, and injection equipment resulting in riskier drug injection practices [7–11]. They result in more dispersed and less safe disposal of used injection equipment [12]. The fear and threat of arrest and searches by police can have a chilling effect on the implementation of emerging harm reduction innovations like drug checking [13]. Though rationalized as public safety actions, sweeps and crackdowns focused on people who use drugs, experience poverty, have marginal housing, and are out in public result in more harm than good [14]. The Centers for Disease Control and Prevention recommend against clearing tent encampments in the absence of available housing due to the risk of disrupting access to medical care [15].

Substance use disorder (SUD) bridge clinics provide low-barrier, on-demand access to medications for opioid use disorder as well as overdose prevention, harm reduction, and infection screening [16–19]. Features of low-barrier models include flexible scheduling and walk-in services, a non-punitive approach to ongoing substance use, decreased stigma about SUD compared to traditional care settings, and incorporation of patient goals and choice into medication decisions. These clinics serve a population at high risk of overdose and infectious complications of injection drug use [16, 20]. While police presence can reduce PWUD’s access to harm reduction services, less is known about how this presence affects utilization of low-barrier SUD treatment services [13, 21].

On August 1, 2019, the Boston Police Department (BPD) initiated Operation Clean Sweep (OCS), which increased police presence in a several-block radius in Boston, MA that contains a county correctional facility, two opioid treatment programs offering methadone, a syringe service program, other outreach-based harm reduction services, a community health center dedicated to the care of people experiencing homelessness, multiple homeless shelters, and an academic medical center with a center for addiction. City officials reported that OCS was a response to the unsafe conditions that had developed in the temporary encampments in the neighborhood, where people who use drugs and experience homelessness had been increasingly concentrated due to criminalization of substance use and limited availability of services in other areas [22]. A violent encounter between an off-duty corrections officer and a community member was cited as an immediate and primary driver of OCS [13]. However, political pressure from residents and businesses in the surrounding neighborhoods had been mounting for months to remove individuals who were experiencing homelessness or congregating in the area, including, but not limited to, people who were intoxicated or using drugs in public [23].

OCS led to thirty-four arrests in its first 2 days, nearly half due to prior warrants and a quarter due to active drug possession [24]. People who use drugs and harm reduction staff reported verbal abuse and physical violence by police officers in addition to consistent requirements to disperse, which displaced people from the neighborhood and resulted in disengagement from social services [13]. Local news reports showed images of personal belongings and wheelchairs being crushed in garbage trucks [25].

As clinical providers in the OCS neighborhood, we heard about these impacts from our patients. We also walked to work past the flashing lights of police cruisers, heard the repeated directives to disperse, and noticed unusually empty streets. We perceived a drop in patient visit volume in our SUD bridge clinic that we hypothesized was due to OCS undermining the low-barrier, on-demand aspect of bridge clinic treatment by rendering the neighborhood less welcoming and unsafe for people who would otherwise have walked in for services. The aim of this study is to systematically examine the association between OCS and service utilization at our SUD bridge clinic.

## Methods

In this retrospective cohort study, we used clinical data from the low-barrier SUD bridge clinic at Boston Medical Center (BMC), a safety-net hospital in the Boston, MA neighborhood where OCS took place, to examine the association between OCS and service utilization. The bridge clinic is a subspecialty addiction setting offering same-day, on-demand access to medications for opioid use disorder, other SUD treatment, and harm reduction services [16]. All bridge clinic patients are seeking care for substance-related problems, and they are referred to long-term care settings after stabilization. We used an interrupted time series design to investigate whether OCS was associated with changes in our primary outcome, the number of daily bridge clinic visits. Interrupted time series is a quasi-experimental approach applied to longitudinal data in consistent intervals to assess for changes in outcomes following an intervention such as OCS [26]. In order to control for secular and seasonal trends in clinic visit volume and allow us to evaluate the effect of the intervention, we compared bridge clinic visit volume to daily General Internal Medicine (GIM) clinic visits as a negative control. The GIM clinic is an academic general primary care practice in the same safety-net facility. Although GIM offers robust SUD treatment, including an office-based addiction treatment program (OBAT) to which the bridge clinic often refers patients after stabilization, it is a general primary care setting and just 7% of GIM patients are estimated to have a SUD [27]. Therefore, we chose GIM patients for comparison because they were not likely to be impacted by OCS but were likely to have been impacted by other local- and institution-level factors such as seasonality or disruption of public transportation that we do not account for in the model. Due to differences in staffing between the bridge clinic and GIM, the model was adjusted for number of provider sessions per day; both practices define one session as 4 hours of clinical care.

We divided the study into a 6-week pre-OCS period (June 20–July 31, 2019), a 2-week period when OCS was active (August 1–August 13, 2019), and a 4-week post-OCS period (August 14–September 11, 2019). Though daily assessments of clinic volume had substantial day-to-day variability, we selected a daily interval to allow for enough observations during the intervention period to assess for changes in visit volumes. We plotted daily clinic visits during the study period and used segmented linear regression to test for changes in the number of bridges and GIM clinic visits before, during, and after OCS. We included terms for baseline trend as well as slope and level changes during the intervention (OCS) and after the intervention (after OCS). A slope change indicates gradual change in the outcome during the assessment period, and level changes indicate immediate changes following an intervention. To prevent biased trends due to inclusion of nonsignificant terms, we selected the model using backward selection, sequentially removing terms with *p* > 0.20 and adjusted for autocorrelation [26]. All analyses were conducted with SAS, Version 9.4 (SAS Institute Inc., Cary, NC, USA). The Boston University Medical Campus Institutional Review Board approved this study as non-human subjects research.

## Results

During the 12 weeks of the study, a total of 608 clinic visits were completed in the bridge clinic and 21,381 in GIM (Table 1). Bridge clinic visits averaged 2.8 per provider/session [standard deviation (SD) 0.9] pre-OCS, 2.0 (SD 0.7) during OCS, and 3.0 (SD 1.4) after OCS. The mean number of GIM clinic visits per provider session before OCS was 7.0 (SD 0.6), during OCS was 6.8 (SD 0.6), and after OCS was 7.0 (SD 0.6).

**Table 1:** Clinic visit volume in the bridge and General Internal Medicine (GIM) clinics

	Pre-OCS	During OCS	Post-OCS	Total
Bridge clinic	307	86	215	608
GIM clinic	11,012	3,768	6,601	21,381

Pre-OCS: June 20–July 31, 2019. During OCS: August 1–August 13, 2019. Post-OCS: August 14–September 11, 2019

**Table 2:** Results of backward selection for interrupted time series parameters of Operation Clean Sweep and the Boston Medical Center bridge clinic visit volume. Boston, MA. June–September 2019

	Baseline				OCS level change				OCS slope change				After OCS level change				After OCS slope change			
	Estimate	Lower 95%	Upper 95%	P value	Estimate	Lower 95%	Upper 95%	P value	Estimate	Lower 95%	Upper 95%	P value	Estimate	Lower 95%	Upper 95%	P value	Estimate	Lower 95%	Upper 95%	P value
Full model	−0.021	−0.038	− 0.004	0.016	0.643	− 0.286	1.573	0.171	− 0.100	− 0.197	− 0.002	0.045	1.442	0.502	2.383	0.003	0.141	0.041	0.241	0.007
Segment regression model results (General Internal Medicine clinics																				
Full model	0.002	− 0.09	0.013	0.697	− 0.066	− 0.701	0.570	0.836	− 0.020	− 0.079	0.040	0.515	0.070	− 0.530	0.670	0.816	0.026	− 0.035	0.087	0.396
Step 1	0.002	− 0.008	0.012	0.738					− 0.024	− 0.064	0.015	0.222	0.095	− 0.463	0.652	0.729	0.031	− 0.006	0.068	0.097
Step 2									− 0.20	− 0.047	0.008	0.166	0.080	− 0.455	0.615	0.765	0.028	− 0.004	0.061	0.088
Step 3									− 0.017	− 0.035	0.002	0.083					0.027	− 0.004	0.059	0.090

In adjusted segmented regression models, there was a small baseline slope decrease in visit volume per provider session at the bridge clinic (-0.021, 95% CI -0.038 to -0.004, *P* = 0.016). During OCS, there was a nonsignificant level increase (0.643, 95% CI -0.286 to 1.573, *P* = 0.171) and significant decrease in slope of visits per provider session (0.100, 95% CI − 0.197 to − 0.002, *P* = 0.045) (Table 2). After OCS, there was a significant level increase (1.442, 95% CI 0.502 to 2.383, *P* = 0.003) and slope increase (0.141, 95% CI 0.041 to 0.241, *P* = 0.007) in bridge clinic visits per provider session. In adjusted segmented regression models for GIM clinics, there was a small, nonsignificant decrease in slope during OCS (-0.017, 95% CI -0.035 to 0.002, *P* = 0.083). After OCS completed, there was a small nonsignificant slope increase in clinic visits per provider session (0.027, 95% CI -0.004 to 0.059, *P* = 0.090).

**Fig. 1:**
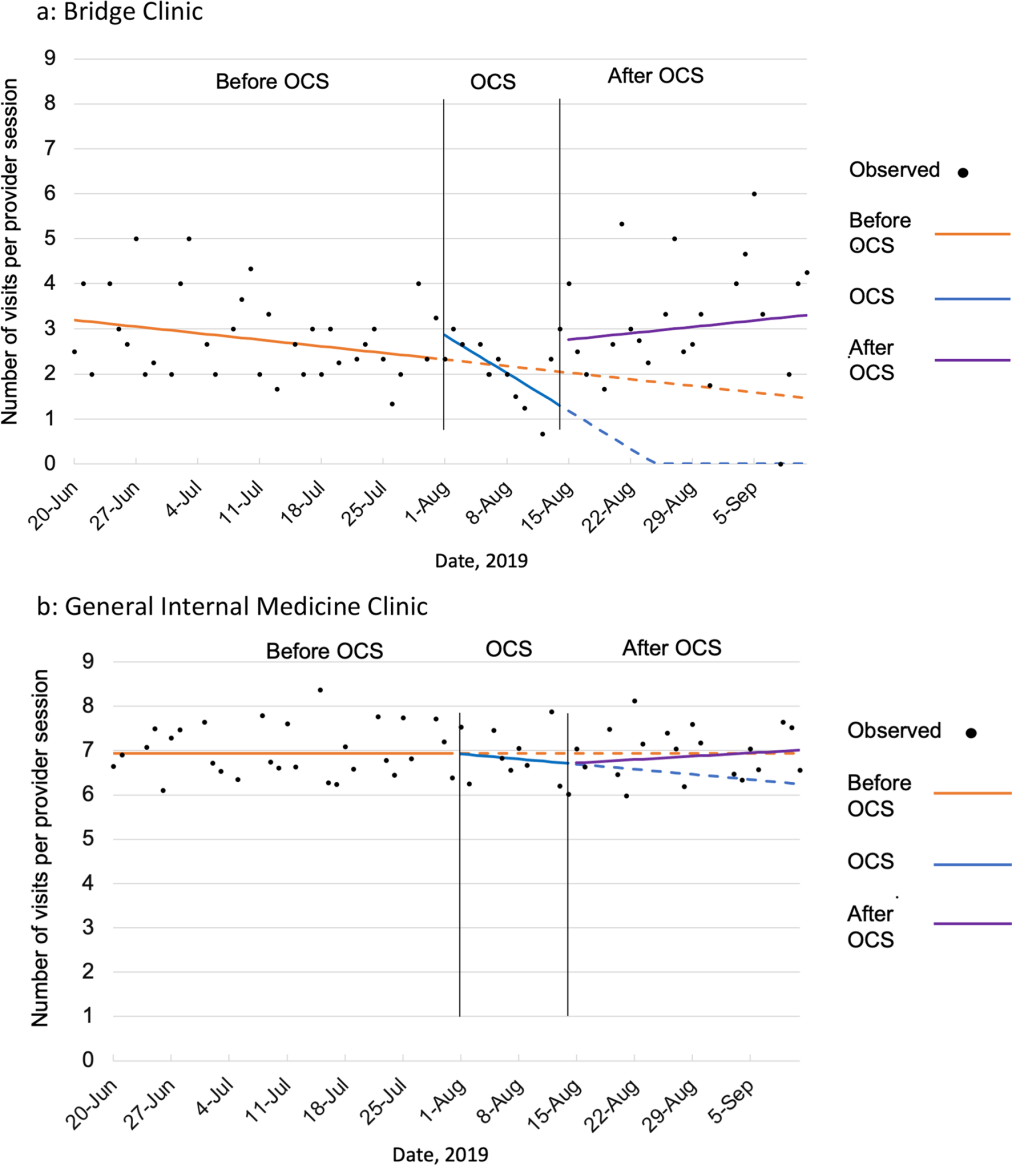
Estimated trends in visit volume at Boston Medical Center’s Bridge Clinic and General Internal Medicine clinic before and after Operation Clean Sweep (OCS). June 20, 2019–September 11, 2019. **A** Bridge Clinic. **B** General Internal Medicine Clinic. Solid lines indicate modeled results based on observed visit volume. Dotted lines indicate estimations of visit volume in each of two projected trend conditions: No OCS (i.e., if OCS had not occurred) and continued OCS (i.e., if OCS continued beyond the observed 2-week period)

Compared to projected number of visits per provider session based on pre-intervention baseline trends, visits decreased from an estimated 2.1 to 1.3 at the end of OCS and increased from 1.3 to 3.3 one month after OCS (Fig. 1), a relative difference of 2 visits per session. Estimated GIM clinic visits per provider session based on the pre-intervention baseline trend were stable at 6.9. Modeled results for GIM visits show decrease to 6.7 per provider session at the end of OCS before returning to 7.0 one month after OCS, nonsignificant changes (Fig. 1).

## Discussion

People who inject drugs and other people with SUD are a highly policed population [9]. This study suggests that a 2-week police action in Boston, MA, was associated with a significant decrease in clinic visits at a low-barrier substance use disorder bridge clinic. We also observed a doubling in volume following the conclusion of OCS, suggesting pent-up demand for bridge clinic services.

Our results add to a growing body of evidence on the consequences of police actions on PWUD. Prior work has demonstrated that PWUD displaced from their usual locations and those who fear police reprisal engage in riskier injection practices including supply sharing and injecting alone, thereby increasing risk of bacterial infections, HIV, viral hepatitis, and death from overdose [7–9, 28]. Contact with police who employ traditional approaches to drug use has also been associated with an increased risk of future overdose fatality [29]. These hardships are disproportionately felt by people experiencing homelessness and people of color, as more resourced PWUD are able to find indoor spaces to inject and people of color are more highly policed [7]. Consistent with other studies, in these data we observed decreased bridge clinic utilization following increased police presence [7, 28, 30]. While our study was not designed to elucidate the specific mechanisms by which OCS led to decreased bridge clinic utilization, in authors’ experience patients reported that incarceration and leaving the area due to fear of incarceration were important factors. Displacement of people from the neighborhood by police, via frequent directives to not to loiter, also likely played a large role [13].

Our finding that OCS was associated with reduced bridge clinic service utilization is concerning for several reasons. Buprenorphine, the primary medication prescribed by the bridge clinic for SUD during the study period, is associated with robust individual and community benefits. People with SUD treated with buprenorphine are less likely to die of opioid overdose or of any other cause and reduce risk behaviors that lead to transmission of infections like HIV [30]. Police actions like OCS designed to reduce the community impact of SUD may have opposite consequences. Future SUD-directed initiatives may be more effective if delivered by medical, behavioral health, and public health teams. If police actions continue, they should mitigate harms on PWUD by fostering and incorporating overdose prevention, other harm reduction services, and engagement in treatment for those who are interested instead of merely increasing the number of incarcerated or displaced persons struggling with addiction. For example, rather than responding to public substance use with arrests, police in Vancouver, Canada, have partnered with public health agencies and refer to harm reduction services or substance use treatment [31].

Our study has limitations. As a single-center analysis, our findings may not be generalizable to other locations or time periods. Further, we were unable to determine if OCS resulted in a compensatory increase in bridge clinic and other addiction service utilization in other Boston neighborhoods or nearby cities. We were unable to adjust for patient-level characteristics which may be associated with visit attendance. Additionally, as OCS occurred over a short time period, we used daily visits per provider session assessments in our modeling approach which increases variability in the data and subsequently the possibility of utilization differences by random chance. The fact that GIM clinic volume remained consistent lends confidence to the interpretation that our findings about changes in bridge clinic visit volume are associated with OCS. However, though we use GIM as a negative control to protect against seasonal variability, we are unable to adjust for differential effects on seasonality in the two clinics. Finally, we use a quasi-experimental design; these are observational data with a high degree of variability which precludes a causal interpretation.

This study found that an operation characterized by increased police presence and arrests was associated with decreased service utilization at a low-barrier SUD clinic. Public health policymakers, law enforcement, and health service providers, including harm reduction and treatment providers, should consider and work to mitigate the unintended harms to access to substance use care when police actions are conducted among people who use drugs. We encourage a broader shift towards funding structural remedies, as the current City of Boston administration has done through the creation of low-threshold transitional housing, to address the upstream drivers of the interconnected crises of homelessness and substance use [32, 33].

## Data Availability

The datasets generated and analyzed during the current study are available from the corresponding author on reasonable request.

## Abbreviations

PWUD: People who use drugs
SUD: Substance use disorder
BPD: Boston Police Department
OCS: Operation Clean Sweep
BMC: Boston Medical Center
GIM: General Internal Medicine
